# CKD in disadvantaged populations

**DOI:** 10.1186/s40697-015-0050-0

**Published:** 2015-04-29

**Authors:** Guillermo Garcia-Garcia, Vivekanand Jha, on behalf of the World Kidney Day Steering Committee

**Affiliations:** Nephrology Service, Hospital Civil de Guadalajara, University of Guadalajara Health Sciences Center, Guadalajara, Jal Mexico; Postgraduate Institute of Medical Education and Research, Chandigarh, India; George Institute for Global Health, New Delhi, India; University of Oxford, Oxford, UK; International Society of Nephrology, Rues de Fabriques 1B, 1000 Brussels, Belgium

*“Of all of the forms of inequality, injustice in health is the most shocking and inhumane.”*

Dr. Martin Luther King, Jr.

March 12, 2015 will mark the 10th anniversary of World Kidney Day (WKD), an initiative of the International Society of Nephrology and the International Federation of Kidney Foundations. Since its inception in 2006, WKD has become the most successful effort ever mounted to raise awareness among decision-makers and the general public about the importance of kidney disease. Each year WKD reminds us that kidney disease is common, harmful and treatable. The focus of WKD 2015 is on CKD in Disadvantaged Populations. This article reviews the key links between poverty and CKD and the consequent implications for the prevention of kidney disease and the care of kidney patients in these populations.

Chronic kidney disease (CKD) is increasingly recognized as a global public health problem and a key determinant of the poor health outcomes. There is compelling evidence that disadvantaged communities, ie, those from low resource, racial and minority ethnic communities and/or indigenous and socially disadvantaged backgrounds, suffer from marked increases in the burden of unrecognized and untreated CKD. Although the entire populations of some low and middle-income countries could be considered disadvantaged, further discrimination on the basis of local factors creates a position of extreme disadvantage for certain population groups (peasants, those living in some rural areas, women, the elderly, religious minorities, etc.). The fact that even in developed countries, racial and ethnic minorities bear a disproportionate burden of CKD and have worse outcomes, suggests there is much to learn beyond the traditional risk factors contributing to CKD-associated complications [1].

About 1.2 billion people live in extreme poverty worldwide. Poverty negatively influences healthy behaviors, health care access and environmental exposure, all of which contribute to health care disparities [2] (Table 1). The poor are more susceptible to disease because of lack of access to goods and services, in particular clean water and sanitation, information about preventive behaviors, adequate nutrition, and reduced access to healthcare [3].

**Table 1:** **Possible mechanisms by which poverty increases the burden of disease**

**Health Behavior**	**Access to Health Care**	**Biological Factors**	**Environmental Factors**
Lack of information on preventive behaviors	Lack of access to health care	Low-birth weight	Increased exposure to pollutants
Lack of knowledge on how best to respond to an episode of illness	Greater distance from health care providers	Genetic predisposition	Increased exposure to communicable diseases
	Lack of out-of-pocket resources	Cummulative biological risk profiles	Lack of clean water sanitation
Health beliefs and unhealthy behaviors		Inadequate nutrition	

## CKD in developed countries

In the US, ethnic minorities have a higher incidence of end stage renal disease (ESRD) Despite similar prevalence rates for early stages of CKD [4], poor outcomes such as ESRD are 1.5-4 times higher [2,5-7] among minorities (ie, African American, Hispanic and Native Americans). Poverty further increases the disparity in ESRD rates, with African-Americans being at greater risk [8]. In the UK, rates of treated ESRD are higher in ethnic minority groups and with increasing social deprivation [9]. Similarly in Singapore, the CKD prevalence is higher among Malays and Indians compared to the Chinese, with socioeconomic and behavioral factors accounting for 70-80% of the excess risk [10].

ESRD incidence is also higher among the less advantaged indigenous populations in developed countries. Canadian First Nations people experience ESRD at rates 2.5-4 times higher than the general population [11]. In Australia, the increase in the number of indigenous people starting renal replacement therapy (RRT) over the past 25 years exceeded that of the non-indigenous population by 3.5 fold, largely due to a disproportionate (>10-fold) difference in ESRD due to type II diabetic nephropathy, a disease largely attributable to lifestyle issues such as poor nutrition and lack of exercise [12]. Indigenous populations also have a higher incidence of ESRD due to glomerulonephritis and hypertension [13]. Compared to the US general population, the ESRD incidence rate is higher in Guam and Hawaii, where the proportion of indigenous people is high, again driven primarily by diabetic ESRD [14]. Native Americans have a greater prevalence of albuminuria and higher ESRD incidence rate [15-18]. Nearly three-quarters of all incident ESRD cases among this population have been attributable to type II diabetes.

## CKD in developing countries

Poverty related factors such as infectious diseases secondary to poor sanitation, inadequate supply of safe water, environmental pollutants and high concentrations of disease-transmitting vectors continue to play an important role in the development of CKD in low-income countries. Although rates of diabetic nephropathy are rising, chronic glomerulonephritis and interstitial nephritis are among the principal causes of CKD in many countries. Of note is the emergence of HIV-associated nephropathy as the major cause of CKD in Sub-Saharan Africa [19].

A high prevalence of CKD of unknown etiology has been reported in rural agricultural communities from Central America, Egypt, India and Sri Lanka. Male farmworkers are affected disproportionately, and the clinical presentation is suggestive of interstitial nephritis, confirmed on renal biopsies. The strong association with farm work has led to suggestions that exposure to agrochemicals, dehydration, and consumption of contaminated water might be responsible [20]. Additionally, the use of traditional herbal medications is common and frequently associated with CKD among the poor [21,22]. In Mexico, CKD prevalence among the poor is 2–3 fold higher than the general population, and the etiology is unknown in 30% of ESRD patients [23-26].

## Low-birth weight and risk of CKD in the disadvantaged populations

An association between low birth weight (LBW) due primarily to nutritional factors and kidney disease has been described in disadvantaged populations. The frequency of LBW is more than double in the Aboriginal population than in the non-Aboriginal population of Australia. The high prevalence of albuminuria in this population has been linked to low nephron number related to low birth weight [27,28]. Morphometric studies of kidney biopsies in the Aboriginals show glomerulomegaly, perhaps secondary to nephron deficiency, which might predispose to glomerulosclerosis [29,30]. A correlation between LBW and CKD has also been described in poor African Americans and Caucasians living in the Southeastern US [31]. Similarly, in an Indian cohort, low birth weight and early malnutrition were associated with later development of metabolic syndrome, diabetes and diabetic nephropathy [32]. The finding of a high prevalence of proteinuria, elevated blood pressure and CKD of unknown etiology in South Asian children may also be explained by this mechanism [33,34].

## Disparities in access to renal replacement therapy

A recent analysis shows that globally, there were 2.6 million people on dialysis in 2010, 93% in high or upper middle-income countries. By contrast, the number of people requiring RRT was estimated at 4.9-9 million, suggesting that at least 2.3 million died prematurely because of lack of access to RRT. Even though diabetes and hypertension increase the burden of CKD, the current provision of RRT is linked largely to two factors: per capita GNP and age, suggesting that poverty is a major disadvantage for receiving RRT. By 2030, the number of people receiving RRT around the world is projected to increase to 5.4 million. Most of this increase will be in developing countries of Asia and Africa [35].

Access to RRT in the emerging world is dependent mostly on the health care expenditures and economic strength of individual countries, with the relationship between income and access to RRT being almost linear in low and middle-income countries [19,36]. In Latin America, RRT prevalence and kidney transplantation rates correlate significantly with gross national income and health expenditure [37], while in India and Pakistan, less than 10% of all ESRD patients have access to RRT [38]. Additionally, developing countries have low transplant rates because of a combination of low levels of infrastructure; geographical remoteness; lack of legislation governing brain death; religious, cultural and social constraints; and commercial incentives that favour dialysis [39].

There are also differences in utilization of renal replacement modalities between indigenous and non-indigenous groups in the developed countries. In Australia and New Zealand, the proportion of people receiving home dialysis is considerably lower among indigenous people. At the end of 2007 in Australia, 33% of non-indigenous people requiring RRT were receiving home-based dialysis therapies, in contrast to 18% of Aboriginal people. In New Zealand, home-based dialysis was utilized by 62% of non-indigenous RRT population but only by 42% of Maori/Pacific Islanders [12]. The rate of kidney transplantation is also lower amongst disadvantaged communities. Maori and Pacific people are only 25% as likely to get a transplant as European New Zealanders, and the proportion of indigenous people who underwent transplantation and had a functioning kidney transplant is lower among Aboriginal Australians (12%) compared to non-indigenous Australians (45%). In the UK, white individuals from socially deprived areas, South Asians and blacks were all less likely to receive a pre-emptive renal transplant or living donor transplants than their more affluent white counterparts [9]. A multinational study found that when compared with white patients, the likelihood of receiving a transplant for Aboriginal patients was 77% lower in Australia and New Zealand, and 66% lower in Canadian First Nations individuals [40].

Disparities in renal care are more evident in developing nations. Data from India shows that there are fewer nephrologists and nephrology services in the poorer states. As a result, people living in these states are likely to receive less care [41]. In Mexico, the fragmentation of the health care system has resulted in unequal access to renal replacement therapy. In the state of Jalisco, the acceptance and prevalence rates in the more economically advantaged insured population were higher (327 pmp and 939 pmp, respectively) than for patients without medical insurance (99 pmp and 166 pmp, respectively) The transplant rate also was dramatically different, at 72 pmp for those with health insurance and 7.5 pmp for those without it [42].

## The bidirectional relationship between poverty and CKD

In addition to having a higher disease burden, the poor have limited access to resources for meeting the treatment costs. A large proportion of patients who are forced to meet the expensive ESRD treatment costs by incurring out-of-pocket expenditure, get pushed into extreme poverty. In one Indian study, over 70% patients undergoing kidney transplantation experienced catastrophic healthcare expenditures [43]. Entire families felt the impact of this, including job losses and interruptions in education of children,

## Outcomes

Overall mortality rates among those who do receive RRT are higher in the indigenous, minorities, and the uninsured populations, even after adjustment for co-morbidities. The hazard ratios for death on dialysis relative to the non-indigenous group are 1.4 for Aboriginal Australians and New Zealand Maori [44]. The Canadian First Nations patients achieve target levels for BP and mineral metabolism less frequently [45]. In the US, living in predominantly black neighborhoods was associated with higher than expected mortality rates on dialysis and increased time to transplantation [46]. Similarly, black patients on PD had a higher risk of death or technique failure compared to whites [47].

In Mexico, the mortality on PD is three-fold higher among the uninsured population compared to Mexican patients receiving treatment in the US, and the survival rate is significantly lower than the insured Mexican population [48], while in India almost two-thirds of the patients are unable to continue dialysis beyond the first 3 months due to financial reasons [49].

## Summary

The increased burden of CKD in disadvantaged populations is due to both global factors and population-specific issues. Low socioeconomic status and poor access to care contribute to health care disparities, and exacerbate the negative effects of genetic or biologic predisposition. Provision of appropriate renal care to these populations requires a two-pronged approach: expanding the reach of dialysis through development of low-cost alternatives that can be practiced in remote locations, and implementation and evaluation of cost-effective prevention strategies. Kidney transplantation should be promoted by expanding deceased donor transplant programs and use of inexpensive, generic immunosuppressive drugs. The message of WKD 2015 is that a concerted attack against the diseases that lead to ESRD, by increasing community outreach, better education, improved economic opportunity, and access to preventive medicine for those at highest risk, could end the unacceptable relationship between CKD and disadvantage in these communities.

Résumé

On attribue l’accroissement du fardeau maladie rénale des populations défavorisées à certains facteurs mondiaux et à des enjeux spécifiques de ces populations données. Un statut socioéconomique faible et une accessibilité aux soins de santé déficiente sont des facteurs qui contribuent aux disparités des soins de santé, et qui aggravent les prédispositions génétiques ou biologiques. L’approvisionnement des populations défavorisées en soins rénaux adéquats nécessite une approche à deux volets : il faut étendre la portée des soins d’hémodialyse par le biais du développement d’initiatives à faible coût pouvant être réalisées en région éloignée, d’une part, et implémenter et évaluer, d’autre part, des stratégies rentables de prévention. La promotion de la transplantation rénale devrait s’effectuer par le développement des programmes de transplantations provenant de personnes décédées, et par l’utilisation de médicaments immunosuppresseurs génériques moins couteux. Le message de la journée mondiale du rein de 2015 est qu’une collaboration accrue des différents professionnels dans le combat des maladies pouvant mener au stade terminal de la maladie rénale pourrait mettre un frein à la relation inacceptable qui perdure entre la maladie rénale chronique et les populations défavorisées, et ce, en augmentant les activités de sensibilisation au sein de ses populations et les chances de développement économique, en améliorant la qualité de l’éducation, et en permettant l’accès aux médicaments de prévention.
